# Potential effect of amniotic fluid-derived stem cells on hyperoxia-induced pulmonary alveolar injury

**DOI:** 10.1186/s13287-022-02821-3

**Published:** 2022-04-04

**Authors:** Amany Solaiman, Radwa A. Mehanna, Ghada A. Meheissen, Soha Elatrebi, Rasha Said, Nahed H. Elsokkary

**Affiliations:** 1 Histology and Cell Biology Department, Faculty of Medicine, Alexandria University, Dr Fahmi Abdelmeguid St., Al. Mowassat Campus, Alexandria, 21561 Egypt; 2 Medical Physiology Department, Faculty of Medicine, Alexandria University, Dr Fahmi Abdelmeguid St., Al. Mowassat Campus, Alexandria, 21561 Egypt; 3 Center of Excellence for Research in Regenerative Medicine and Its Applications CERRMA, Faculty of Medicine, Alexandria University, Alexandria, Egypt; 4 Clinical Pharmacology Department, Faculty of Medicine, Alexandria University, Dr Fahmi Abdelmeguid St., Al. Mowassat Campus, Alexandria, 21561 Egypt; 5 Medical Biochemistry Department, Faculty of Medicine, Alexandria University, Dr Fahmi Abdelmeguid St., Al. Mowassat Campus, Alexandria, 21561 Egypt

**Keywords:** Amniotic fluid-derived mesenchymal stem cells, Hyperoxia, Pulmonary tissue fibrosis, Histological structure, Pathological and functional changes, Protective effects

## Abstract

**Background:**

With the widespread of Coronavirus Disease 2019 pandemic, in spite of the newly emerging vaccines, mutated strains remain a great obstacle to supportive and preventive measures. Coronavirus 19 survivors continue to face great danger of contacting the disease again. As long as no specific treatment has yet to be approved, a great percentage of patients experience real complications, including among others, lung fibrosis. High oxygen inhalation especially for prolonged periods is per se destructive to the lungs. Nevertheless, oxygen remains the first line support for such patients. In the present study we aimed at investigating the role of amniotic fluid-mesenchymal stem cells in preventing versus treating the hyperoxia-induced lung fibrosis in rats.

**Methods:**

The study was conducted on adult albino rats; 5 pregnant female rats were used as amniotic fluid donors, and 64 male rats were randomly divided into two groups: Control group; where 10 rats were kept in normal atmospheric air then sacrificed after 2 months, and hyperoxia-induced lung fibrosis group, where 54 rats were exposed to hyperoxia (100% oxygen for 6 h/day) in air-tight glass chambers for 1 month, then randomly divided into the following 5 subgroups: Hyperoxia group, cell-free media-treated group, stem cells-prophylactic group, stem cells-treated group and untreated group. Isolation, culture and proliferation of stem cells were done till passage 3. Pulmonary function tests, histological examination of lung tissue under light and electron microscopes, biochemical assessment of oxidative stress, IL-6 and Rho-A levels, and statistical analysis of data were performed. F-test (ANOVA) was used for normally distributed quantitative variables, to compare between more than two groups, and Post Hoc test (Tukey) for pairwise comparisons.

**Results:**

Labelled amniotic fluid-mesenchymal stem cells homed to lung tissue. Stem cells administration in the stem cells-prophylactic group succeeded to maintain pulmonary functions near the normal values with no significant difference between their values and those of the control group. Moreover, histological examination of lung tissues showed that stem cells-prophylactic group were completely protected while stem cells-treated group still showed various degrees of tissue injury, namely; thickened interalveolar septa, atelectasis and interstitial pneumonia. Biochemical studies after stem cells injection also showed decreased levels of RhoA and IL-6 in the prophylactic group and to a lesser extent in the treated group, in addition to increased total antioxidant capacity and decreased malondialdehyde in the stem cells-injected groups.

**Conclusions:**

Amniotic fluid-mesenchymal stem cells showed promising protective and therapeutic results against hyperoxia-induced lung fibrosis as evaluated physiologically, histologically and biochemically.

**Supplementary Information:**

The online version contains supplementary material available at 10.1186/s13287-022-02821-3.

## Background

In the worldwide Coronavirus Disease 2019 (COVID-19) pandemic, supportive therapies including respiratory care for affected patients, especially in more severe cases, are employed [1]. Before the emergence of vaccination, hospitalized patients with COVID-19 developed worsening pneumonia and acute respiratory distress syndrome (ARDS) with high rates of mortality. Patients with severe disease often need oxygenation support with high-flow oxygen (O_2_) and non-invasive positive pressure ventilation, but the safety of these measures is uncertain. High concentrations of O_2_ may be needed for prolonged periods that might exceed 2 weeks [2]. Prolonged sub-lethal O_2_ exposures probably end up with lung fibrosis [3]. Patients worldwide who have survived the pandemic continue to battle various symptoms of the illness, known as post-COVID syndrome, which may vary from mild fatigue and body aches to severe forms requiring long term O_2_ therapy and lung transplantation due to lung fibrosis [4].

O_2_ also remains the first-line therapy used for many other acute and chronic respiratory diseases for hypoxemic patients. However, target goals for normoxemia are not well defined. Therefore, iatrogenic hyperoxia is a very common situation [5]. Supra-physiological levels of O_2_ are toxic to the tissues due to exacerbated reactive oxygen species (ROS) production and impairing the homeostasis of multiple cellular processes. Cell injury caused by high O_2_ levels activates inflammatory cascades, mononuclear cellular infiltration, this is in addition to the oedema that amplifies the tissue damage. The lung is the first—but not the only- organ affected by hyperoxia [6]. The alveolar epithelial and alveolar capillary endothelial cells are vulnerable targets for ROS injury caused by it [7]. The acute lung injury following hyperoxia was thoroughly investigated [5], yet the chronic sequelae still need to be discovered.

Amniotic fluid (AF) is an attractive source of mesenchymal stem cells (MSCs) for therapeutic applications. It can be safely collected during or at the end of gestation, with no ethical concerns and low risk of tumorigenicity [8]. AF-MSCs exhibit low immunogenicity and high anti-inflammatory properties. They are able to self-renew, highly proliferative, and have a broad differentiation potential, making them amenable for cell-based therapies [9].

## Material and methods

### Aim of the study

In this study, we aim to investigate the chronic sequelae of hyperoxia on lung alveolar structure and respiratory functions. Additionally, exploring the possible protective and therapeutic role of AF-MSCs on hyperoxia-induced pulmonary alveolar injury.

### Experimental animals

The study was conducted on Sprague–Dawley adult albino rats; 5 pregnant female rats were used as amniotic fluid donors, and Sixty-four male rats weighing 150–200 g, 6–8 weeks of age were used for the study. Rats were purchased from the Medical Physiology department experimental animal facility, Alexandria Faculty of Medicine, where they were housed under a 12–12 h light–dark cycle at 25 °C with food and water provided ad libitum. All experiments were conducted in accordance with the approved guidelines set by the Research Ethics Committee of Alexandria Faculty of Medicine, Egypt (IRB N^o^: 00012098- FWA N^o^: 00018699). Membership through Alexandria University in International Council of Laboratory Animal Science organization ICLAS http://iclas.org/).

### Isolation, culture and proliferative capacity of AF-MSCs

The whole procedure of isolation and characterization was performed at the Center of Excellence for Research in Regenerative Medicine and its Applications (CERRMA), Alexandria Faculty of Medicine.

On day 14 of pregnancy, the female rats were euthanized by an overdose of the inhalation anesthesia. The uterus was dissected, and each gestational sac was opened from the anti-mesenteric side to avoid bleeding from the placental site. Needles of 30-gauge calibration were used to aspirate AF from each sac separately [10]. The AF was then spun at 1400 × g for 5 min to pellet the cells. Pelleted cells were cultured in 60 mm culture dish where 5 ml of low glucose Dulbecco's Minimum Essential Medium (DMEM, Lonza) supplemented with 10% foetal bovine serum (FBS, Sigma Aldrich) and 1% penicillin/streptomycin (Lonza) were added. Cells were incubated at 37 °C and 5% CO_2_ in a humidified incubator and observed daily under a phase contrast inverted microscope. The media was changed every 2–3 days. Cells were passaged after reaching 80% confluence using 0.25% (w/v) trypsin/ethylene diamine tetra acetic acid (EDTA) (Lonza), then cultured in T75 cm^2^ flasks [11]. Cell viability was assessed using the trypan blue (Gibco) exclusion test, and cells were counted using a Neubauer hemocytometer [12, 13].

### Characterization of AF-MSCs

#### Immunophenotyping of AF-MSCs using flow cytometer

Cells were characterized using fluorescent-labelled monoclonal antibodies (mAb) for CD45, CD90, and OCT4 markers. Cells at passages 3–5 were trypsinized with 0.25% trypsin–EDTA solution, washed with phosphate-buffered saline (PBS), counted and incubated at room temperature for 30 min in the dark, with monoclonal phycoerythrin (PE)-conjugated antibody for CD45 (Abcam, ab23396, UK), monoclonal fluorescein isothiocyanate (FITC)-conjugated antibody for CD90 (Anti-Thy1.1) (Abcam, ab225, UK), and the monoclonal FITC-conjugated antibody for OCT4 antibody (Abcam, ab181557, UK). Immunofluorescence on the viable cells was analyzed using Becton Dickinson, FACS caliber flow cytometer equipped with Cell Quest software [12, 14].

#### Colony-forming unit assay

The colony-forming potential of the cultured cells at passage 3 (P3) was tested. In this assay, 500 cells were plated on a 35 mm culture dish in complete media and incubated for 14 days. The cells were then fixed and stained using Crystal Violet (Sigma-Aldrich, USA) at 3% (w/v) in methanol for 5 min at room temperature. The stain was removed, and cells were washed with distilled water. The number of colonies was counted and the colony-forming potential was calculated as follows: Plating efficiency = the number of colonies formed/number of cells plated × 100. All visible colonies were counted; the number of colonies displaying five or more cells was scored under the phase-contrast inverted microscope. A CFU potential of over 40% was considered to be optimal for AF-MSCs culture [15].

#### Differentiation of AF-MSCs into adipocytes, osteocytes and chondrocytes

Osteogenic differentiation; Cells were plated in an osteogenic differentiation medium containing L-DMEM, 10% FBS, 0.1 μM dexamethasone (Sigma-Aldrich), 200 μM L-ascorbic acid-2-phosphate (Sigma-Aldrich) and 10 mM β-glycerol phosphate (Sigma-Aldrich) for 21 days and induction was confirmed by Alizarin Red S staining.

Adipogenic differentiation; cells were plated in an adipogenic differentiation media containing 1 μM dexamethasone, 58 μg/ml insulin, 0.5 mM 3-isobutyl-1-methylxanthine (IBMX), and 200 μM indomethacin, for 2 weeks then cells were treated with insulin medium (Differentiation medium, containing 10 μg/ml insulin). Medium was changed with fresh insulin medium every 2 days for one week and induction was confirmed by Oil Red O staining.

Chondrogenic differentiation was done using StemXVivoVR Chondrogenic Base Media (R&D Systems, USA) and StemXVivo Chondrogenic Supplement (R&D Systems, USA). 2.5 × 10^5^ P3 MSCs were re-suspended in chondrogenic-differentiation media then centrifuged at 200×*g* for 5 min in a conical falcon tube. The tube’s cap was loosened to allow gas exchange and was incubated upright at 37 °C and 5% CO_2_. Every 3 days, the media was changed, and after 28 days, the chondrogenic spheroidal pellet was harvested and stained for assessing the cartilage-specific proteoglycan using Alcian blue 8GX stain [16].

#### AF-MSCs labelling and tracking

For tracking the injected cells, the cytoplasm/cell membrane of cells prior injection were labelled with a fluorescent probe (chloromethylbenzamido octadecyl indocarbocyanines) (CM-DiI) (molecular probes, Thermo Fisher USA) [17]. Cell Tracker CM-Dil was supplied as 50 µg/vial to be reconstructed in 50 µl dimethyl sulfoxide (DMSO) in a concentration of 1 µg/µl as a stock solution. After the manufacturer protocol; 7 µl of Dil were used to label 1 × 10^6^ cells in 7 ml PBS and incubated in 37 °C for 7 min. After labelling, cells were washed with PBS and re-suspended in fresh medium for IV injection in rats. The rats were then sacrificed 72 h after injection for visualizing the labelled cells in lung tissue under confocal microscopy (Leica microsystems, DMi8, Germany) [18].

### Establishment of hyperoxia

Rats were housed in airtight glass chambers 66Lx47Wx43H and exposed to chronic intermittent hyperoxia (100% O_2_ concentration 6 h/day for 30 days) with O_2_ flow rates of 10 L/min and pressure = 1 bar.

### Experimental design

Sixty-four male rats were randomly divided into the following groups:

Control group (CG), where 10 rats were kept in normal atmospheric air O_2_ then sacrificed after 2 months. Hyperoxia-induced lung fibrosis group, where fifty-four rats were exposed to the chronic intermittent hyperoxia, then randomly divided into the following subgroups:

- Hyperoxia group (HG); 10 rats were sacrificed after 1 month of hyperoxia exposure for the assessment of hyperoxia-induced lung fibrosis model.
- Cell-free media-treated group (CFM-TG); 10 rats received I.V. injection of 1 ml cell-free complete media after induction, then sacrificed after another month.
- Stem cells-prophylactic group (SC-PG); 12 rats received I.V. injection of AF-MSCs (1 × 10^6^ cells in 1 ml complete media) [19], 48 h after exposure to hyperoxia then the exposure was continued for the rest of the month. 10 rats were then sacrificed after another month for assessment, while 2 rats were sacrificed 72 h after injection with labelled stem cells for assessing stem cells homing.
- Stem cells-treated group (SC-TG); 12 rats received I.V. injection of AF-MSCs (1 × 10^6^ in 1 ml complete media). 10 rats were sacrificed after another month for assessment, while 2 rats were sacrificed 72 h after injection with labelled stem cells for assessing stem cells homing.
- Untreated group (Unt-G); 10 rats left untreated for another month after induction of fibrosis, then sacrificed for assessment.

### Pulmonary functions

Tidal volume (VT), minute respiratory volume (MRV) and forced vital capacity (FVC) were assessed using a PowerLab digital data acquisition system at the beginning and the end of the study. The ventilatory parameters were recorded using a pneumotachometer MLT1L (Lab chart 8, AD Instruments, Castle Hill, NSW, Australia) with P1 channel end connected to the outlet of the NP/Whole Body Plethysmography [20].

### Lung tissue histological processing

At the end of the experiment, all rats were sacrificed and both lungs were dissected. The left lung was kept frozen at − 80 °C for later biochemical study. While the lower right lobe of each right lung was divided into two halves; one half was fixed in 10% neutral-buffered formalin, then processed to obtain (5–6 µm) thin sections, which were routinely stained with H&E and Masson’s trichrome for light microscopic (LM) examination [21] using (Olympus BX41, Tokyo, Japan) equipped with a spot digital camera (Olympus DP20, Tokyo, Japan). Histomorphometric study was done, using NIH Fiji program (NIH, Bethesda, MD, USA), where the area percentage of collagen fibers in Masson’ trichrome stained sections, the inter-alveolar septal thickness and the alveolar surface area in H & E stained sections, were measured in five randomly selected sections for each item. Data was presented as mean ± standard deviation (SD) of randomly selected ten fields/section (n = 5/group [22]. The other half was cut into smaller parts (0.5–1 mm^3^) and immediately fixed in 3% phosphate buffered glutaraldehyde pH 7.4, then processed to obtain ultra-thin sections [23] for transmission electron microscope (TEM) examination (Jeol 1400 plus Tokyo, Japan).

### Biochemical assessment

#### Assessment of Ras homolog family member A (RhoA) and interleukin-6 (IL-6) expression in lung tissue using western blot [24]

Western blot was used to assess RhoA and IL-6 expression in lung tissue. Lung tissues were homogenized and the tissue lysate was prepared by adding radio-immunoprecipitation cell lysis (RIPA) buffer (Cat. No. AR0105, Bosterbio), Tris (PH 8.0) and protease inhibitor (Cat. No. AR1182, Bosterbio). The lysates were assayed for total protein concentration by Lowry method [25], and stored until analyzed. After protein electrophoresis, protein transfer from sodium dodecyl sulphate polyacrylamide gel onto nitrocellulose membrane was performed by electro blotting. Following transfer, bands were detected using Polyclonal Anti-RhoA Antibody (Cat. No. YPA2321, Biopsies Co), Anti IL-6 Polyclonal Antibody (Cat. No. YPA1248, Biopsies Co.) and AntiBeta Actin (Cat. No. BPA1012, Biopsies Co). Next, the membranes were incubated with the rabbit IgG DAB Chromogenic Reagent Kit (Cat. No. SA2020, Bosterbio). Protein relative band densities ratio were assessed using NIH Fiji program (NIH, Bethesda, MD, USA) and protein expression was normalized against β-actin.

#### Measurements of biomarkers of oxidative stress

The lung tissues were homogenized in 1–2 ml of 5 mM cold potassium phosphate buffer (pH 7.4), centrifuged at 4000 rpm for 15 min at 4 °C and the supernatant was removed and stored at − 80 °C for assay of total antioxidant capacity (TAC) (Cat. No. TA 25 13, Biodiagnostics) and malondialdehyde (MDA) (Cat. No. MD 25 29, Biodiagnostics).

##### Measurement of TAC

The determination of the antioxidant capacity is performed by the reaction of antioxidants in the sample with a defined amount of exogenously provided hydrogen peroxide (H_2_O_2_). The antioxidants in the sample eliminate a certain amount of the provided H_2_O_2_. The residual H_2_O_2_ is determined calorimetrically at 510 nm by an enzymatic reaction which evolves the conversion of 3,5, dichloer-2-hydroxybenzenesulphonate to a colored product [26].

##### Measurement of MDA

Lipid peroxidation is determined as thiobarbituric acid which reacts with MDA in acidic medium to form thiobarbituric reactive product measured calorimetrically at 534 nm [27].

### Statistical analysis of data

Data was fed to the computer and analysed using IBM SPSS software package version 20.0 (Armonk, NY: IBM Corp). Qualitative data were described using number and percent. The Kolmogorov–Smirnov test was used to verify the normality of distribution Quantitative data were described using range (minimum and maximum), mean, standard deviation and median. Significance of the obtained results was judged at the 5% level. F-test (ANOVA) was used for normally distributed quantitative variables, to compare between more than two groups, and Post Hoc test (Tukey) for pairwise comparisons [28].

## Results

### Characterization of AF-MSCs

#### Morphological characterization

**Fig. 1:**
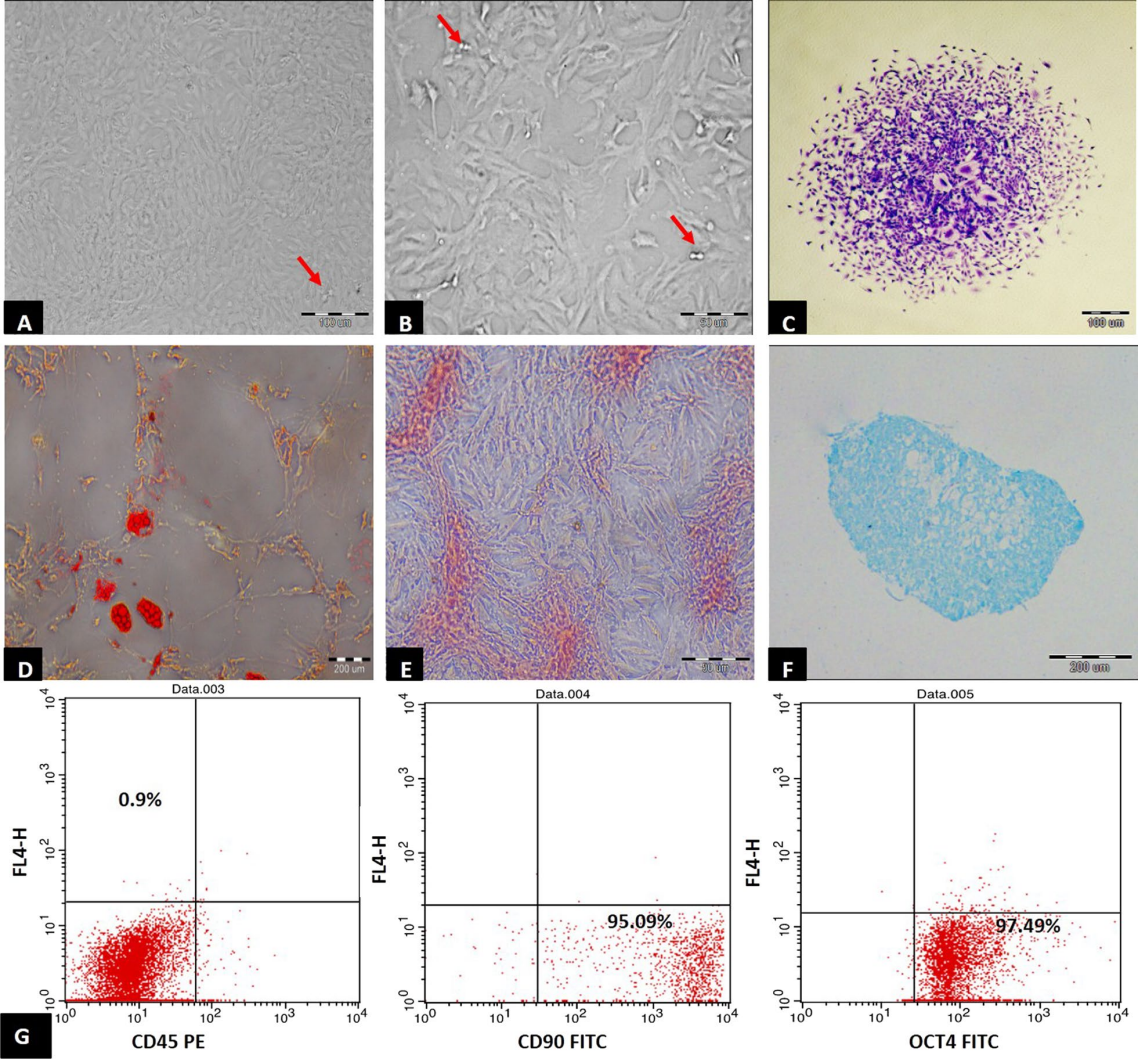
Phenotypic characterization of AF-MSCs. **A**, **B** Phase contrast inverted microscope, Morphological characteristics of AF-MSCs showing **A** passage 3, 90% confluent spindle fibroblast-like cells (× 100, scale bar 100 µm). **B** Passage 3, 80% confluent spindle fibroblast-like cells forming monolayer (× 200, scale bar 50 µm), rounded cells undergoing mitotic division, proliferating cells (red arrows). **C** CFU assay, P3 Crystal Violet stain showing one colony (× 100, scale bar 100 µm). **D** Differentiated cells into adipocytes showing positive red staining of fat droplets using Oil red stain (× 40, scale bar 200 µm). **E** differentiated cells into osteoblasts showing positive red staining of calcium ions with Alizarin red stain (× 200, scale bar 50 µm). **F** differentiated cells into chondrocytes showing positive blue staining of cartilage-specific proteoglycans using Alcian blue stain (× 40, scale bar 200 µm). **G** Flow cytometric analysis of cell-surface markers of AF-MSCs at passage 3, cells were negative for the CD45 hematopoietic marker, upper left quadrant showing only 0.9% (upper scatter), 95.09% of the cultured cells expressed the mesenchymal cell marker CD90 in lower right quadrant (middle scatter) and 97.49% of cells showed pluripotent cell marker OCT4 in lower right quadrant (lower scatter). *n* = 10 in all groups

The cell cultures were monitored daily using phase-contrast inverted light microscope. In primary culture (P0), cells were small and rounded, then became spindle in shape after 72 h of culture, displaying a heterogeneous population and reached 70–80% confluence in approximately 10–12 days. With passaging, cell growth was accelerated and cell morphology changed, exhibiting large, flattened and spindle-shaped cells. Mitotic rounded cells appeared, demonstrating proliferation. At P3 the culture represented a homogenous fibroblast-like cell monolayer (Fig. 1A, B).

#### Colony forming unit-fibroblast (CFU-F) assays

After 5–7 days of incubation, cells gradually proliferated into small colonies. Two weeks post-seeding, large colonies were seen with crystal violet staining (Fig. 1C). The colony-forming assay showed that each dish with 100 cells gave 93% ± 1.35 of colonies after 14 days.

#### Immunophenotyping of AF-MSCs by flow cytometry

FACS analysis for AF-MSCs at P3 showed that 95.09% of the cultured cells expressed the mesenchymal multipotent CD90 surface marker. 97.49% of cells expressed the pluripotent surface marker OCT4, while they were almost negative for the CD45 hematopoietic marker shown only in 0.9% of cells (Fig. 1G).

#### Differentiation of AF-MSCS

AF-MSCs were successfully differentiated into adipocytes, osteocytes and chondrocytes-like cells noted with the characteristic positive staining of fat droplets with Oil red stain (Fig. 1D), Ca^2+^ deposits with Alizarin stain (Fig. 1E) and collagen-specific proteoglycans with Alcian blue (Fig. 1F), respectively.

### Homing of AF-MSCS into lung tissue

**Fig. 2:**
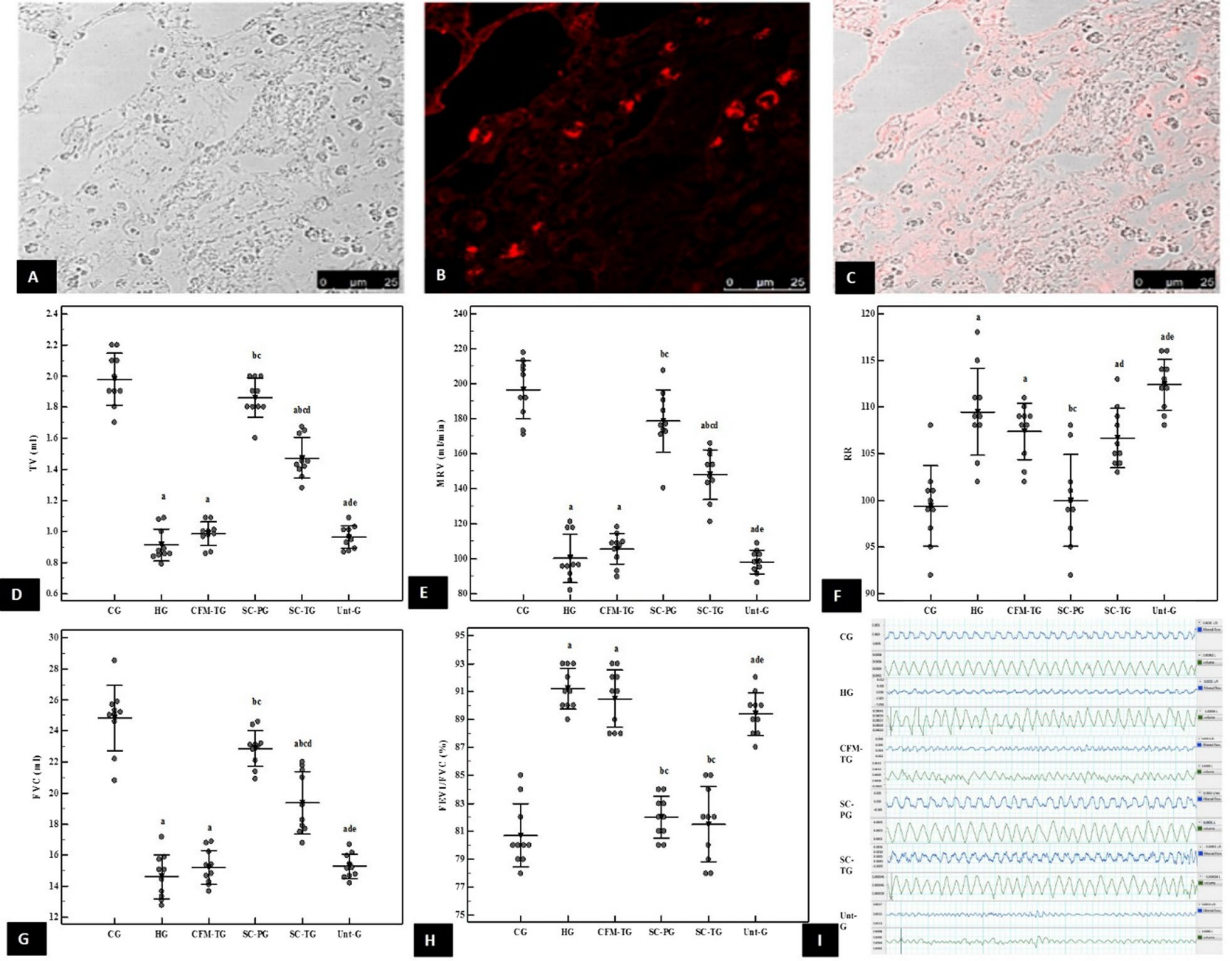
Confocal Microscopic images of lung tissue (**A**–**C**). DIC image (**A**). Labelled AF-MSCs (red fluorescence) homed on alveolar wall (**B**). Merged DIC and fluorescent images (**C**) scale bar 25 mm. Representative graphs of mean values of TV, MRV, RR, FVC and FEV1/FVC% respectively (**D**–**H**). a; significant compared to CG. b: significant compared to HG. c: significant compared to CFM-TG. d: significant compared to SC-PG. e: significant compared to SC-TG. Pairwise comparison bet. Each 2 groups using Post Hoc Test (Tukey). Significance at *p* ≤ 0.05. Error bars represent S.E.M. *n* = 10 in all groups. (**I**) Representative tracings of airway flow (upper) and tidal volume (lower) in each panel of the different studied groups. Air flow and tidal volume were decreased in HG, CFM-TG, Unt -G. Treatment with stem cells improved both parameters in SC-PG and to a lesser extent in SC-TG. Abbreviations: DIC; differential interference contrast. CG; control group, HG; Hyperoxia group, CFM-TG; Cell-free media-treated group, SC-PG; Stem cells-prophylactic group, SC-TG; Stem cells-treated group, Unt-G Untreated group, TV; tidal volume, MRV; minute respiratory volume, RR; respiratory rate, FVC; forced vital capacity, FEV1; forced expiratory volume 1

Labelled AF-MSCs were tracked under the laser scanning confocal microscope in lung tissue 72 h after injection. Red fluorescent cells were seen in the lung tissue (Fig. 2A–C).

### Pulmonary function assessment

The HG showed a significant decrease in MRV, FEV1 and FVC by 50%, 30% and 40% and an increase in RR and FEV1/FVC by 10% & 13% compared to the CG (*p* < 0.001) demonstrating that the induced model had affected the pulmonary functions. The CFM-TG and Unt-G showed no significant improvement in pulmonary function tests as compared to HG and showed a 46% and 50% decrease in the MRV, 30% in FEV1 and 40% and 36% in FVC compared to the CG respectively. An increase in RR by 11% and FEV1/FVC by 12% in these two groups (CFM-TG and Unt-G) as compared to CG (*p* < 0.001) further proved that pulmonary functions remained deteriorated with no significant improvement whether spontaneously Unt-G or when treated with CFM. However, stem cells administration in the SC-PG succeeded to maintain MRV, FEV1, FVC, FEV1/FVC as well as RR near the normal values with no significant difference between their values and those of the CG. Moreover, treatment with stem cells after one month of O_2_ therapy in the treated group increased the MRV, FEV1 as well as FVC by 50%, 14%, and 33% in comparison to the HG, and decreased RR by 3% yet this improvement did not reach normal levels as there was significant difference between their values and those of the CG (*p* < 0.001) (Fig. 2).

### Histological assessment

#### Haematoxylin and eosin (H&E) stain

**Fig. 3:**
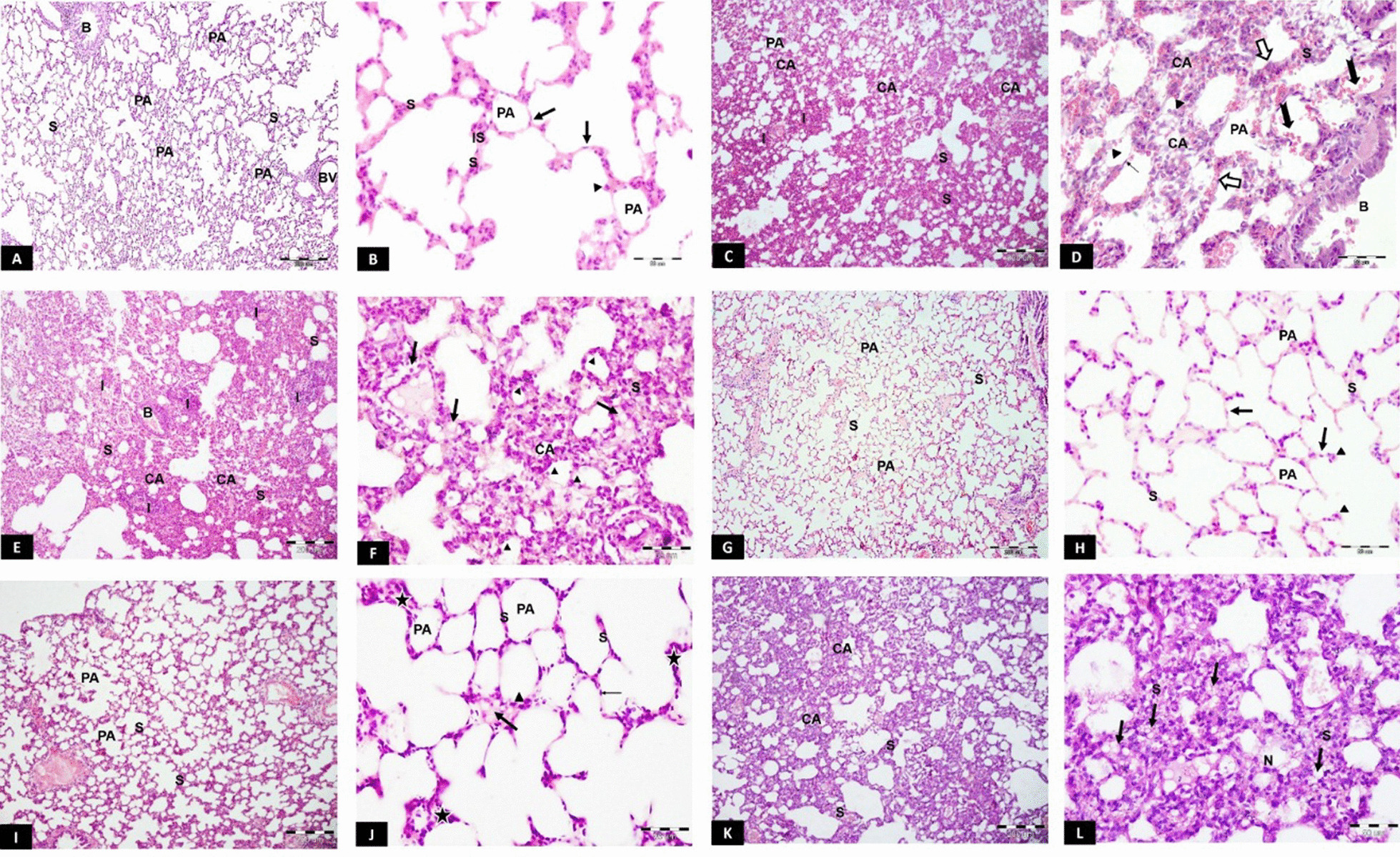
H&E results. **A**, **B** Light photomicrographs of CG showing: **A** Patent alveoli (PA) and thin classical interalveolar septa (S). **B** folded bronchiolar epithelium, BV; blood vessel. **B** Alveoli lined with flat squamous type I pneumocytes (arrow) and bulging cuboidal type II cells (arrowhead). Thin interalveolar septa (S) with only few interstitial cells (IS) are also seen. **C**, **D** light photomicrographs of HG revealing: **C** Most alveoli are collapsed (CA) with thick interalveolar septa (S) and cellular infiltration (I). PA; a patent alveolus. **D** Collapsed alveoli (CA), thickened septa (S) and extravasated red blood corpuscles in the alveolar lumen (notched black arrow) and in the septa (white arrow). Few type I pneumocytes (thin arrow) and many type II pneumocytes (arrowhead) are also seen. **B** bronchiole. **E**, **F** light photomicrographs of CFM-TG showing: E: Collapsed alveoli (CA), thick septa (S) and cellular infiltration (I). **B** a bronchiole with desquamated cells in its lumen. **F** Thickened septa (S) with multiple interstitial cells having vacuolated cytoplasm (arrow). Most of the alveolar lining cells have rounded nuclei (arrowhead). **G**, **H** Light photomicrographs of SC-PG showing: **G** Thin interalveolar septa (S) separating the patent inflated alveoli (PA). **H** Flat type I pneumocytes (arrow) and large bulging eosinophilic type II (arrow head). S; thin interalveolar septum, PA; a patent alveolus. **I**, **J** light photomicrographs of SC-TG showing: **I** Mildly thickened Interalveolar septa (S) between the patent alveoli (PA). **J** Some septa are thin (S), others are mildly thickened (star). Some interstitial cells show vacuolation (thick arrow). Flattened type I (thin arrow) and few type II (arrowhead) are also seen. **K**, **L** light photomicrographs of Unt-G showing: **K** Collapsed alveoli (CA) and thickened interalveolar septa (S). **L** Thick interalveolar septa (S) showing vacuolated interstitial cells (arrow). Most of the cells show deeply-stained nuclei (N). H&E stain, scale bar (**A**, **C**, **E**, **G**, **I**, **K**) 200 µm, (**B**, **D**, **F**, **H**, **J**, **L**) 50 µm. *n* = 10 in all groups

Light micrographs of the CG revealed normal lung architecture, patent alveoli and thin interalveolar septa. The alveoli were lined with flat squamous type I pneumocytes and bulging cuboidal type II (Fig. 3A, B). HG showed atelectasis, thickened interalveolar septa with interstitial pneumonia, congested blood vessels and red blood corpuscles extravasation in the alveolar lumen and the septa. Few type I pneumocytes were seen. On the other hand, many type II pneumocytes with their rounded nuclei were further depicted (Fig. 3C, D). The CFM-TG showed microscopic changes similar to the HG, with collapsed alveoli, thick septa with multiple interstitial cells having vacuolated cytoplasm. Most nuclei lining the alveoli were rounded together with perivascular cellular infiltration (Fig. 3E, F). The SC-PG showed almost normal appearance of the lung architecture with thin interalveolar septa and patent alveoli. Flat squamous type I pneumocytes and bulging eosinophilic type II were also seen (Fig. 3G, H). The SC-TG revealed mostly patent alveoli. The interalveolar septa were mildly thickened and some interstitial cells showed vacuolation (Fig. 3I, J). The Unt-G depicted an appearance similar to the HG with atelectasis and thickened interalveolar septa containing vacuolated interstitial cells. The alveoli were mostly lined with deeply-stained nuclei (Fig. 3K, L).

#### Masson’s trichrome stain

**Fig. 4:**
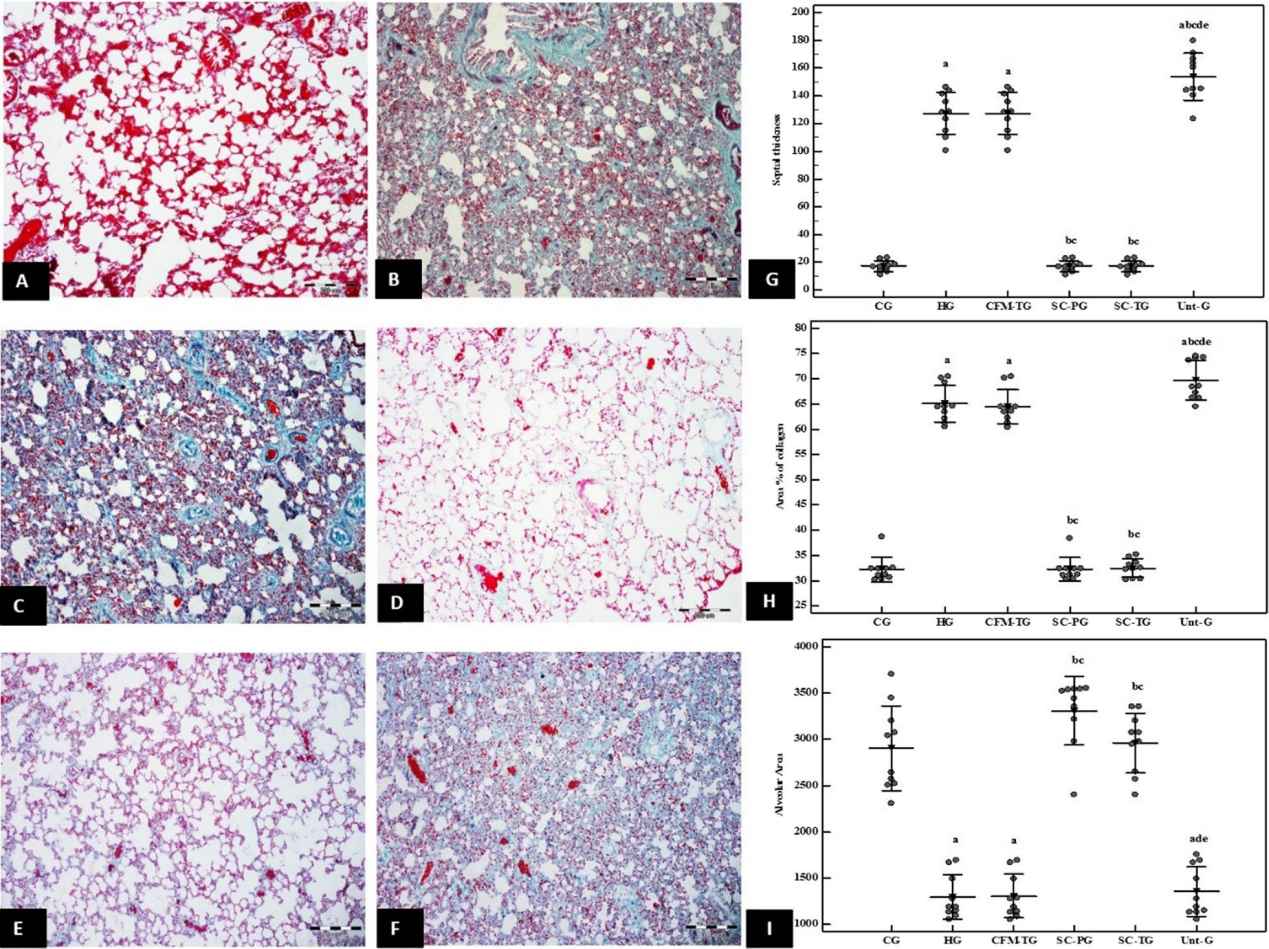
**A**–**F** Masson’s trichrome light photomicrographs. **A** Photomicrograph of the CG showing normal distribution of green-stained collagen fibers in the inter-alveolar septa, perivascular and peribronchiolar. **B** HG showing excessive deposition of collagen fibers in inter-alveolar septa, perivascular and peribronchiolar. **C** CFM-TG showing apparent increase in green-colored areas within the inter-alveolar septa. **D** SC-PG showing limited and focal distribution of green-colored areas within the inter-alveolar septa. **E** SC-TG showing apparent limited distribution of green-colored areas within the inter-alveolar septa and perivascular. **F** Unt-G showing moderate to severe green-colored areas within the inter-alveolar septa. Masson's trichrome, scale bar (**A**–**F**) 200 µm. **G**–**I** Morphometric analysis and Statistical comparison between the studied groups according to: **G** Mean of septal thickness **H** Mean area% of collagen **I** Mean of alveolar area. a: Significant with CG, b: Significant with HG, c: Significant with CFM-TG, d: Significant with SC-TG, e: Significant with Unt-G. Values represent mean ± SD. Statistical significance was determined using ANOVA Test. Pairwise comparison between each 2 groups was done using Post Hoc Test (Tukey). Statistically significant at *p* ≤ 0.05. Error bars represent S.E.M. *n* = 10 for all groups. Abbreviations: CG; control group, HG; Hyperoxia group, CFM-TG; cell-free media treated-group, SC-PG; stem cell-prophylactic group, SC-TG; stem cell- treated group, Unt-G; untreated group. *n* = 10 in all groups

Photomicrographs of the CG showed normal collagen distribution in the interalveolar septa, perivascular and peribronchial. (Fig. 4A). On the other hand, HG showed excessive deposition of collagen (Fig. 4B). Similarly, CFM-TG showed an apparent increase in stained areas of collagen deposition within the interalveolar septa (IAS) (Fig. 4C). On the other hand, SC-PG revealed limited and focal distribution of collagen stained areas within the IAS (Fig. 4D). Likewise, SC-TG showed limited stained areas within the IAS and perivascular as compared to CG (Fig. 4E). Contrarily, the Unt-G depicted moderate to severe stained areas within the IAS, indistinguishable from the HG.

#### Morphometric analysis

##### Morphometric analysis of trichrome-stained sections

The Morphometric analysis of trichrome-stained sections was performed by calculating the percentage area of greenish blue-stained collagen. There was a significant increase in the percentage area of collagen deposition in HG and CFM-TG (*p* < 0.001). On the other hand, SC-PG and SC-TG showed a significant decrease in the area percentage of collagen deposition in comparison to HG, reaching the control levels (*p* < 0.001). Moreover, Unt-G showed statistically significant increase in area percentage of collagen deposition in comparison to CG, SC-PG and SC-TG, with no significant difference to that seen in HG (Fig. 4G).

##### Morphometric analysis of the alveolar surface area

To assess the degree of alveolar collapse, the alveolar surface area was measured morphometrically. There was a significant decrease in surface area of alveoli in HG and CFM-TG versus the CG (*p* < 0.001). On the other hand, SC-PG and SC-TG showed a significant increase in the surface area of the alveoli in comparison to HG. These results were almost the same shown in CG (Fig. 4H).

##### Morphometric analysis of interalveolar septal thickness

The interalveolar septal thickness was similarly measured to evaluate the degree of interruption of blood-air barrier. SC-PG and SC-TG showed no significant difference in inter-alveolar septal thickness in comparison with CG. Yet, there was a significant decrease of septal thickness in comparison to HG (*p* < 0.001) (Fig. 4I).

#### Electron microscopic results

**Fig. 5:**
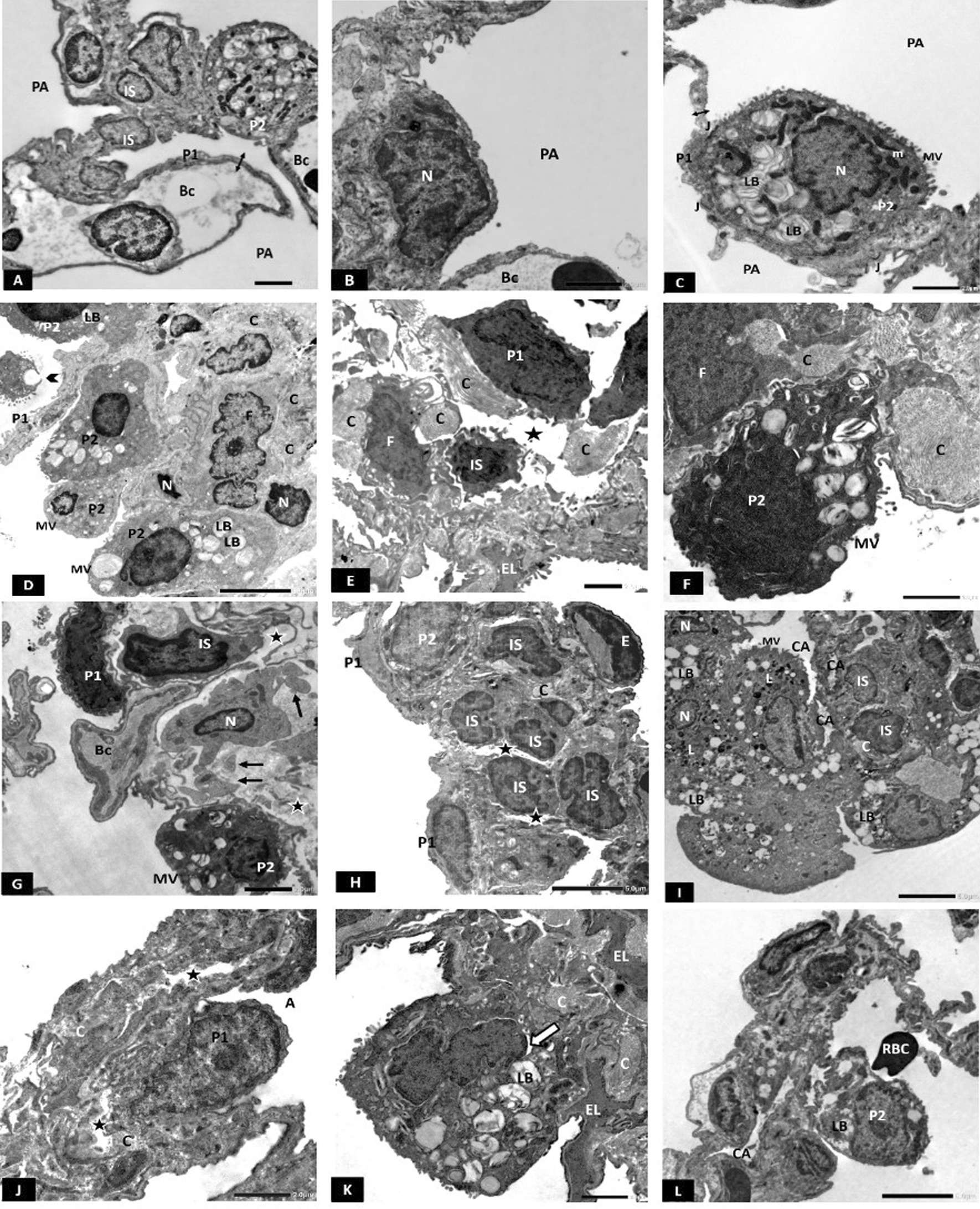
**A**–**C** Electron photomicrographs of the CG showing: **A** Interalveolar septum with few interstitial cells (IS) and patent alveoli (PA) lined by flatted type I (P1) and club-shaped type II pneumocyte (P2). Double arrow; thin blood-air barrier. **B** Type I pneumocyte showing flattened euchromatic nucleus (N). Bc; blood capillary, PA; patent alveoli. **C** Type II pneumocyte (P2) showing prominent microvillous border (MV), euchromatic nucleus (N), mitochondria (m) and multiple lamellar bodies (LB). Intercellular junction (J) is seen between the two types of pneumocytes. Notice the thin blood-air barrier (double arrow). PA; patent alveoli. Scale bar **A**–**C** 2 µm. **D**–**G** Electron photomicrographs of HG showing **D** The IAS is obviously thickened, fibroblasts (F) surrounded by collagen (C) and some interstitial cells have small heterochromatic nuclei (N) were also seen. Multiple type II pneumocytes (P2) with almost empty lamellar bodies (LB) and absent microvilli (MV) are seen. Notice a desquamated type II pneumocyte into the alveolar lumen (arrowhead). P1; type I pneumocyte. **E** Type I pneumocyte (P1) with irregular heterochromatic nucleus and dense cytoplasm. F; fibroblast surrounded by collagen fibrils (C). EL; elastic fibers, IS; interstitial cell (star); interstitial oedema. **F** Type II pneumocyte (P2) with an irregular heterochromatic nucleus and blunted microvillus border (MV). Extensive collagen deposition (C) in the vicinity of a fibroblast (F) is also seen. **G** Type I pneumocyte (P1) with irregular heterochromatic nucleus. Type II pneumocyte (P2) showing heterochromatic nucleus and blunted microvillous border (MV). A cell in the process of apoptosis with multiple apoptotic bodies (arrow) and peripheral nuclear chromatin clumps (N) is also seen. BC; blood capillary, IS; interstitial cell, star; interstitial oedema. Scale bar **D** 5 µm (E, F, G) 2 µm. **H**–**L** Electron photomicrographs of CFM -TG showing: H: Multiple ISCs showing irregular heterochromatic nuclei, and some surrounded with collagen fibrils (C). P1; type I pneumocyte, P2; type II pneumocyte, E; endothelial cell, star; interstitial oedema. **I** Multiple type II pneumocytes having small irregular nuclei (N) and empty lamellar bodies (LB). Many interstitial cells (IS) are also seen. C; collagen. MV; microvilli. CA; collapsed alveolus. **J** Type I pneumocyte with irregular surface. A; alveolus, C; collagen, star; interstitial oedema. **K** Type II pneumocyte with irregular heterochromatic nucleus and mildly dilated perinuclear cisterna (arrow). LB; lamellar body. C; Collagen, EL; elastic fibers. **L** Type II pneumocyte (P2) having empty lamellar body (LB). CA; Collapsed alveolus, RBC; an extravasated red blood corpuscle into the alveolar lumen. Scale bar **H**, **I** 5 µm, **J**–**L** 2 µm. *n* = 10 in all groups

Electron micrographs of CG lungs showed thin interalveolar septum (IAS) with few interstitial cells (ISCs) (Fig. 5A). Type I pneumocytes had flattened euchromatic nuclei (Fig. 5B). Type II pneumocytes showed central euchromatic nucleus, prominent microvillous border, multiple lamellar bodies, and mitochondria (Fig. 5A, C). Intercellular junctions were observed between the two cell types (Fig. 5C). Blood-air barrier (BAB) appeared thin (Fig. 5A, C).

On the other hand, the HG depicted variable degenerative changes in the lung parenchyma. The IAS was obviously thickened with multiple ISCs having heterochromatic nuclei (Fig. 5D–F) and fibroblasts with collagen (Fig. 5D–F) and elastic fibers deposition (Fig. 5E). In addition to obvious interstitial oedema (Fig. 5E, G). Type I pneumocytes appeared having irregular heterochromatic nuclei and electron-dense cytoplasm (Fig. 5E, G). Type II pneumocytes had blunted microvilli, vacuolated cytoplasm, and abnormal, sometimes empty lamellar bodies (Fig. 5D, E, G). Many type II pneumocytes were seen extruded into the alveolar lumina (Fig. 5D). Apoptotic bodies and peripheral nuclear chromatin clumps were often seen (Fig. 5G).

Electron micrographs of CFM-TG revealed a picture similar to that of the HG. The IAS was also thickened with multiple ISCs showing irregular heterochromatic nuclei (Fig. 5H, I) and collagen deposition (Fig. 5H, J, K). Degenerating type I pneumocytes with irregular surface (Fig. 5J), numerous type II pneumocytes having irregular heterochromatic nuclei (Fig. 5I, K). Some had dilated perinuclear cisternae (Fig. 5K). Many of them contained empty small lamellar bodies (Fig. 5I, L) or large vacuoles (Fig. 5I). Extravasated RBCs in the alveolar lumina (Fig. 5L) that were mostly collapsed (Fig. 5I, L).

**Fig. 6:**
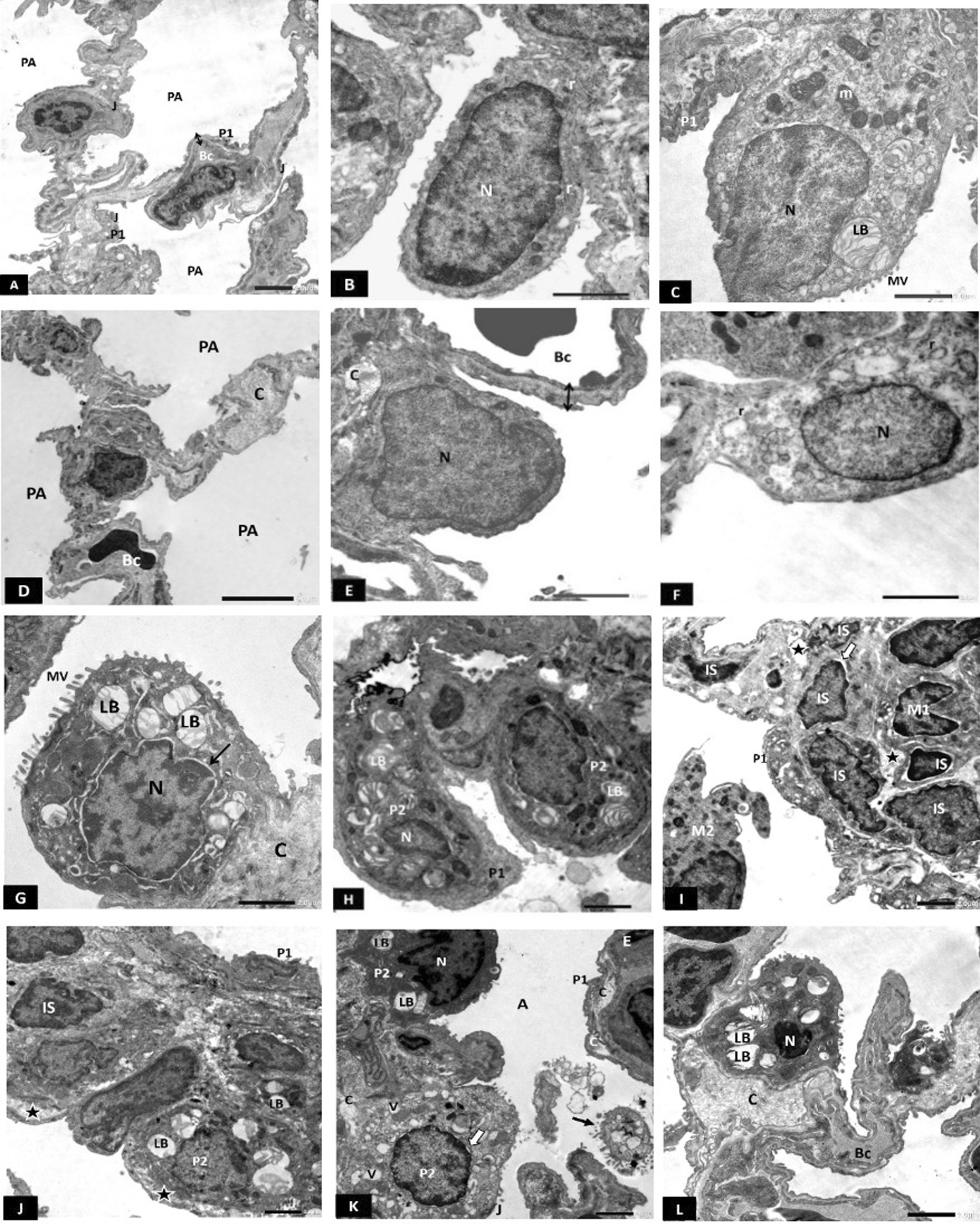
**A**–**C** Electron photomicrograph of SC-PG showing: **A** Thin blood-air barrier (double arrow), patent alveoli (PA) and intact intercellular junctions (J). Bc; blood capillary. P1; type I pneumocyte. **B** Type I pneumocyte showing large euchromatic nucleus (N) and few profiles of rough endoplasmic reticulum cisternae (r). **C** Large bulging type II pneumocyte with characteristic microvilli (MV), lamellar body (LB) and mitochondria (m). P1; type I pneumocyte. Scale bar **A**–**C** 2 µm. **D**–**H** Electron photomicrographs of SC-TG showing: **D** Thin interalveolar septum with few collagen fibrils (C). PA; patent alveolus, Bc; blood capillary. **E** Type I pneumocyte showing slightly bulging euchromatic nucleus (N). Thin BAB can be seen (double arrow). Bc; blood capillary. C; collagen. **F** Type I pneumocyte showing euchromatic flattened nucleus (N) and dilated profiles of rough endoplasmic reticulum cisternae (r). **G** Type II pneumocyte showing many lamellar bodies (LB) and a microvillus border (MV). Arrow: mildly dilated perinuclear cisterna. C; collagen, N; nucleus. **H** Two interstitial type II pneumocytes (P2) showing lamellar bodies (LB). A small nucleus is seen in one of them (N). P1; type I pneumocyte. Scale bar **D** 5 µm, **E**–**H** 2 µm. **I**–**L** Electron photomicrographs of Unt-G showing: **I** Thick interalveolar septum with many interstitial cells (IS) having irregular small nuclei, some with mildly dilated perinuclear cisterna (white arrow). A macrophage with its characteristic kidney-shaped nucleus (M1) is depicted. Another macrophage is seen in the alveolar lumen (M2) with many lysosomes (L) and pseudopodia (PD). Notice interstitial oedema (star). **J** Thick interalveolar septum containing interstitial cells (IS) with irregular heterochromatic nuclei. Type I pneumocyte (P1) with an irregular heterochromatic nucleus and type II pneumocyte (P2) with empty lamellar body (LB) are also seen. Notice interstitial oedema (star). **K** A type II pneumocyte showing dense cytoplasm (P2), an irregular dense nucleus (N) and few lamellar bodies (LB). Another type II pneumocyte (P2) shows many cytoplasmic vacuoles (V) and mildly dilated perinuclear cisterna (white arrow). A third type II pneumocyte is extruded into the alveolar lumen (black arrow). P1; Type I pneumocyte with underlying collagen (C). J; intercellular junction. **L** Type II pneumocyte with heterochromatic small nucleus (N) and empty lamellar bodies (LB). Bc; blood capillary, C; Collagen. Scale bar **I**–**L** 2 µm. *n* = 10 in all groups

Electron micrograph of SC-PG showed marked preservation of alveolar ultrastructure with patent alveoli, thin BAB and intact intercellular junctions (Fig. 6A). Type I pneumocyte had large euchromatic nuclei and few rough endoplasmic reticulum cisternae (Fig. 6B). Large bulging type II pneumocytes showed their characteristic microvilli and typical lamellar bodies. Intercellular junctions were kept intact.

Electron micrograph of SC-TG showed marked improvement of alveolar ultrastructure. Thin interalveolar septum containing few collagen, and patent alveoli were the prominent features (Fig. 6D). Thin BAB can be seen (Fig. 6D, E). Type I pneumocytes showed flattened euchromatic nuclei. However, some showed large slightly bulging nuclei (Fig. 6E, F respectively). Moreover, dilated rough endoplasmic reticulum cisternae were depicted in few type I pneumocytes (Fig. 5E). Type II pneumocytes showed their distinctive microvillus border, and numerous well-formed lamellar bodies (Fig. 6G, H). However, some type II pneumocytes showed small nuclei with peripheral chromatin clumps (Fig. 6H) and dilated perinuclear cisternae (Fig. 6G). Moderate amounts of collagen could be depicted in the IAS (Fig. 6G).

Electron micrograph of Unt-G revealed thickened interalveolar septa with many interstitial cells having irregular small nuclei with peripheral chromatin clumps (Fig. 6I, J), and dilated perinuclear cisternae (Fig. 6I). Interstitial oedema was obvious (Fig. 6I, J). Alveolar and interstitial macrophages were also depicted with their numerous lysosomes and pseudopodia (Fig. 6I). Type I pneumocytes with irregular heterochromatic nuclei and type II pneumocytes with fused lamellar bodies were also seen. (Fig. 6J). Some type II pneumocytes appeared with electron-dense cytoplasm, folded irregular heterochromatic nuclei and few almost empty lamellar bodies (Fig. 6K, L) Some showed dilated perinuclear cisternae (Fig. 6I, K), many cytoplasmic vacuolations (Fig. 6K) and still others were extruded into the alveolar lamina. (Fig. 6K).

### Biochemical assessment

#### Western blot results for RhoA and IL-6 expression in lung tissue

**Fig. 7:**
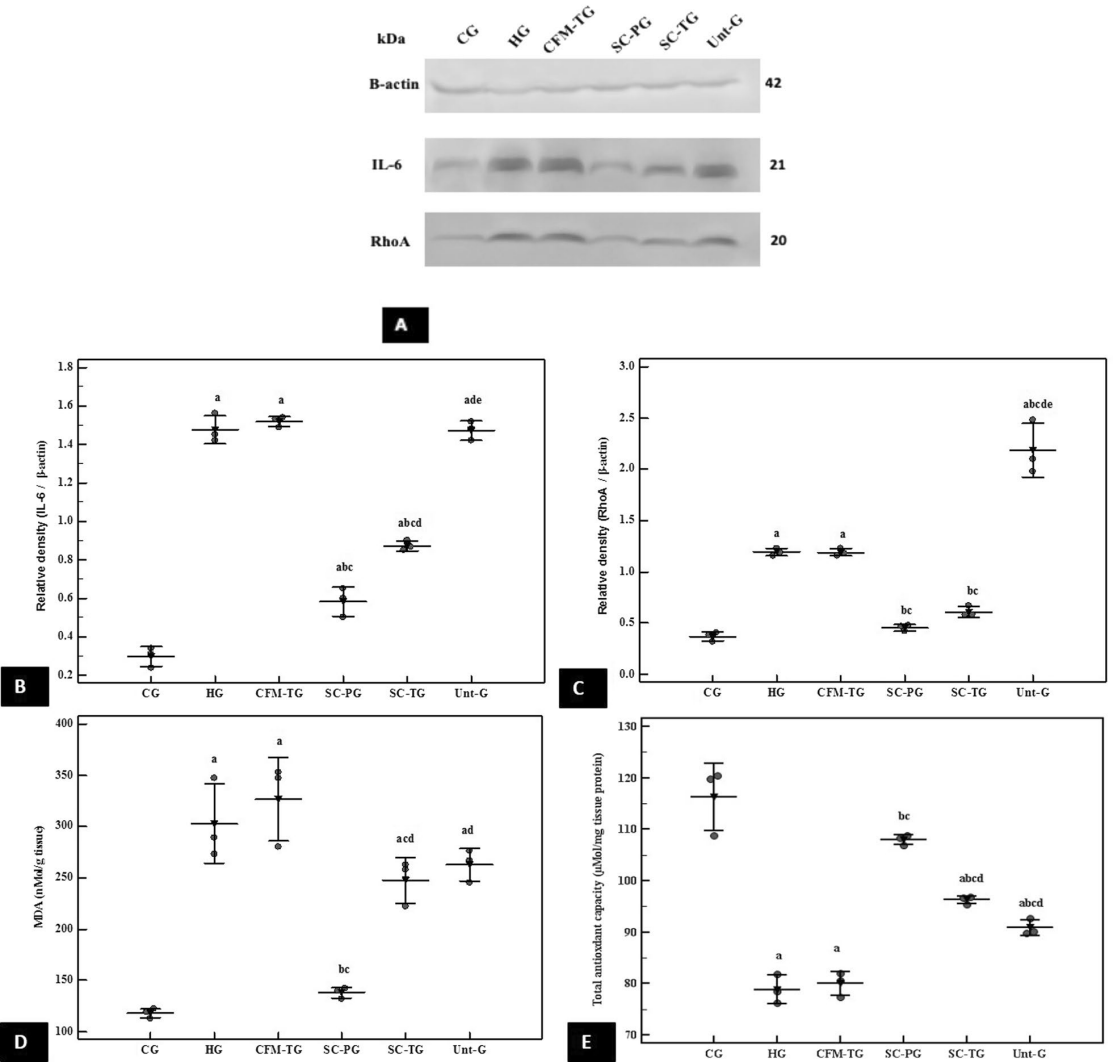
Effect of stem cell treatment on biochemical markers. **A** Western blot analyses of IL-6 and RhoA in lung tissue in all groups. β actin was used for normalization. Images of the bands where obtained from the same gel with no cropping in between the bands. Full length blots are included in the Additional file 1 Fig. S1. **B**, **C** Representative graphs for relative expression of IL-6 and RhoA respectively. **D**, **E** Representative graphs for quantification of MDA and TAC in lung tissue of all experimental groups. Data is represented in mean. *n* = 3 in all groups. a; significant compared to CG. b: significant compared to HG. c: significant compared to CFM-G. d: significant compared to SC-PG. e: significant compared to SC-TG. Pairwise comparison bet. Each 2 groups using Post Hoc Test (Tukey). Significance at *p* ≤ 0.05. Error bars represent S.E.M. Stem cell treatment significantly reduced IL-6, RhoA and increased TAC, while stem cell prophylaxis significantly reduced MDA. Abbreviations: CG; control group, HG; Hyperoxia group, CFM-G; Cell-free media-treated group, SC-PG; Stem cells-prophylactic group, SC-TG; Stem cells-treated group, Unt-G Untreated group, IL-6; interleukin 6, RhoA; Ras homolog family member A, MDA; malondialdehyde, TAC; total antioxidant capacity

RhoA was significantly increased in HG and CFM-TG compared to the CG While it was significantly decreased in the SC-PG and SC-TG compared to HG and CFM-TG (*p* < 0.001). Its value in Unt-G was significantly increased compared to all studied groups. IL-6 was significantly increased in HG, CMF-TG and Unt-G compared to the CG, while it was significantly decreased in the SC-PG and SC-TG compared to HG and CFM-TG (*p* < 0.001) (Fig. 7A–C). Full length blots are included in the Additional file 1 Fig. S1.

#### Oxidative state assessment

The study showed that MDA was significantly increased in HG and CMF-TG compared to the CG, while it was significantly decreased in the SC-PG compared to HG and CFM-TG (*p* < 0.001). On the other side, TAC was significantly decreased in HG and CFM-TG groups than in the CG, while it was significantly increased in SC-PG, SC-TG and Unt-G compared to HG and CFM-TG (*p* < 0.001). Moreover, it showed no significant difference in CG compared to SC-PG, thus reaching normal levels (Fig. 7D, E).

## Discussion

Although O_2_ therapy is the first line of treatment for many hypoxaemia-related diseases, its elaborate and uncontrolled administration can do more harm than good to the patient's condition. In the present study, we implemented our experimental design to induce lung fibrosis upon the already proved model of hyperoxic lung injury. The effects of hyperoxia are assumed to be time and dose dependent [29]. So, unlike most earlier studies, which used continuous short-term hyperoxia [30, 31], this study used intermittent prolonged hyperoxia for the induction of lung fibrosis. The study tracked the structural, physiological and biochemical long-term sequelae of this model of induced lung fibrosis. In addition, it explored the role of therapeutic versus prophylactic AF-MSCs administration on the injured lung alveoli.

Lipid peroxidation of polyunsaturated fatty acids produce a series of complex compounds, including MDA. Therefore, the determination of MDA is widely used as an indicator in evaluating oxidative stress [32]. In this experiment, hyperoxia was coupled with a surge in the MDA, it was significantly higher in HG and CFM-TG, suggestive of increased oxidative stress and lipid peroxidation in these groups. On the other hand, TAC, one of the important indices reflecting the antioxidation of the body, significantly decreased in lung tissues by hyperoxia, indicating the collapse of the antioxidant system.

With the induction of this oxidative burst, mainly by activated inflammatory cells, the secretion of proinflammatory chemokines and cytokines by resident macrophages and epithelial cells is followed [33]. So in the present study, the hyperoxia-induced lung injury was accompanied by pulmonary inflammatory responses as evidenced by increased IL-6. Strong correlation may exist between Il-6 expression and pulmonary injury/inflammation as it poses a strong proinflammatory effect [34].

These findings were further confirmed with the histological picture of the lung in these groups. Where the light micrographs of CFM-TG were indifferent from the HG; both showed alveolar and vascular congestion, diffuse atelectasis with alveolar damage and collapse, intense cellular infiltration denoting interstitial pneumonia. Thickened interalveolar septa with deposition of interstitial collagen fibers were the worst of all changes. So with the large amounts of ROS generated from mitochondrial electron transport or NADPH oxidase-catalyzed reactions during hyperoxia [3], damaged mitochondrial membranes become more permeable for pro-apoptotic components which then pass to the cytoplasm and contribute to the excess ROS. Subsequently, interstitial pulmonary oedema and impaired gas exchange by means of alveolar collapse and disintegration of the alveolar-capillary barrier occur [35]. This was obviously reflected on the rats’ pulmonary functions and thus their mortality rates which was higher in these groups (8%). By the end of the study, surviving animals appeared sick, fatigued and irritable with significant deterioration of pulmonary functions represented by a decrease in MRV, FEV1 and FVC and an increase in RR and FEV1/FVC compared to the CG, SC-PG and SC-TG.

Mach et al. demonstrated that the first structure appeared to be affected by hyperoxia is the vascular endothelial cell [21], and probably this was the cause of capillary congestion and massive RBCs extravasation into the alveoli as well as in the IAS that were commonly encountered in our histological sections in both HG and CFM-TG. Recruitment of immune cells to the lung following oxidative stress, is another cause of increased IAS thickness. This in turn leads to loss of pulmonary barrier function, producing more inflammation and pulmonary oedema that are characteristics of hyperoxic lung injury [31]. Also, According to Kallet et al. [36], after prolonged exposure to a toxic O_2_ tension, the alveolar cells become hyperplastic and hypertrophied, causing a great increase in the thickness of the alveolar walls. These changes persist even after removal to normal O_2_ tensions as demonstrated in the Unt-G.

During lung injury, type II pneumocytes can proliferate and differentiate into type I cells to compensate for damaged cells [37]. When the delicate homeostatic balance is disturbed by oxidative stress, damage of nucleic acids especially in the proliferating type II pneumocytes, might occur. Moreover, damage of proteins, and lipids leading to changes in membrane permeability and fluidity, progressing to lytic damage and cell death [35]. Furthermore, several proteomic investigations have demonstrated significant changes in cellular migration, differentiation, and proliferation proteins in type II pneumocytes following hyperoxic injury [38].

Although resident fibroblasts and circulating progenitors can certainly contribute to the hyperplasia of fibroblasts and the subsequent pathology, an additional source for pulmonary fibroblast formation might be through the differentiation of type II pneumocytes through the process of epithelial-to-mesenchymal transition (EMT) [30]. EMT is a biologically important process involved in tissue remodeling during organogenesis, but the reversion of terminally differentiated epithelium to its mesenchymal origin has been implicated in organ fibrosis [30].

The electron micrographs of HG and CFM-TG revealed variable degenerative changes, confirming the LM results, where type I pneumocytes were infrequently encountered. This can be attributed to the direct toxic effect of hyperoxia on these cells [39]. HG as well as the CFM-TG showed marked hypertrophy of type II pneumocytes with their rounded nuclei, on the expense of the flat type I. This is in line with other authors’ findings who proved the morphological change of type II to type I pneumocytes as a mechanism to compensate for their loss; probably through an intermediate cell stage that may express markers from both cell types [40].

The presence of vacuoles of different sizes were a prominent feature observed in both HG and CFM-TG under the EM in the cytoplasm of interstitial cells but also in the pneumocytes. They were also noticed in the SC-TG and the Unt-G. Presence of vacuoles is generally thought to represent degenerative changes [41]. For example, misfolding of endoplasmic reticulum (ER) proteins by the oxidative environment, leads to ER stress with distended and pinched off vesicles and tubules. Moreover, accumulation of triglycerides within the damaged mitochondria or within the cytoplasm, decreased lipid transport and their accumulation in the cytoplasm, all these changes would manifest as vacuolization [41].

Finally, our EM results in HG, CFM-TG and Unt-G showed prominent lung fibrosis, that probably was an ultimate consequence of pulmonary O_2_ toxicity. Similarly, Hemnes et al. showed in their study that excessive production and deposition of extracellular matrix proteins, e.g., collagen-I, was the most important feature of pulmonary fibrosis in hyperoxia-induced lung injury [39].

In harmony with the fibrosis that was encountered in the EM results, the small GTPase RhoA, that is essential in the regulation of various cellular functions including formation of F-actin stress fibers and focal adhesion complexes and transcription of genes containing serum response element [42] was significantly overexpressed in HG and CFM-TG [30]. This is consistent with the study showing that ROS-dependent RhoA activation is responsible for the increase in collagen-I synthesis in hyperoxic lung fibroblasts of mouse lungs [43].

MSCs derived from amniotic tissue have characteristics of both adult and embryonic stem cell, they express the embryonic cell marker OCT4, thus they possess a higher regenerative power than adult sources. In addition to their immunomodulatory, anti-inflammatory and antioxidant functions, AF MSCs lack the ethical concerns for their use, unlike embryonic stem cells [44].

Devine and colleagues attempted to study the different targets of systemic MSCs injection. In their study, after intravenous (IV) injection of MSCs into rats’ tails, most of the cells were trapped in lung and only a minor fraction of the cells (less than 3%) engrafted in other tissues [45]. Moreover, Chang et al. demonstrated in two of their studies on bronchopulmonary dysplasia, that the IV MSCs administration route is as effective as the intra-tracheal one [46, 47]. This helped our team to confidently rely on the IV route of injection in the present study. Indeed, AF-MSCs were shown in the lung tissue 72 h after their injection further confirming our IV injection choice.

In our study, the administration of stem cells prior to induction of fibrosis in the SC-PG helped in maintaining the pulmonary function tests within normal values. Moreover, their administration after the hyperoxia-induced fibrosis helped to ameliorate the pulmonary functions’ deterioration. This was supported by the significantly decreased MDA and increased TAC levels denoting the maintenance in the redox oxidation balance, together with a reduction in IL-6 level in the lungs denoting an inhibition of pro-inflammatory cytokine production, as well as a reduction in the level of RhoA and these were consistent with relevant studies [3, 48]. Moreover, the histological results went in line with the previous results suggesting the prevention of hyperoxia-induced fibrosis in the lung with stem cells preconditioning as well as slight improvement of the already formed fibrosis in SC-TG.

The biochemical reduction of IL-6 levels suggested that the therapeutic effects of MSCs might be partially mediated through the inhibition of pro-inflammatory cytokine production. Relating our results with the COVID-19 lung destruction, that appears to be associated with a cytokine storm with an increased IL-6 level. This anti-inflammatory effect of MSCs can be a tool to oppose the IL-6 together with other pro-inflammatory mediators and thus attenuate the vigorous inflammatory response that might end up with fibrosis and pulmonary failure. While the mild improvement seen in the Unt-G, can be explained by the natural antioxidant capacity inside the cell.

The prophylactic as well as the therapeutic powers of AF-MSCs can be attributed mainly to the paracrine functions of MSCs; through releasing extracellular vesicles or exosomes; transferring miRNAs to nearby endothelial and epithelial cells, thereby promoting angiogenesis and alveolar repair [49]. According to Antounians et al., compared to bone marrow MSCs, AF-MSCs exosomes were enriched for miRNAs that are critical for lung development, such as the miR17 ~ 92 cluster and its paralogues (miRs-93, -106, -250, and -363). Moreover, their miRNAs that have previously been reported as dysregulated in hypoplastic lungs, such as miR-33 and miR-200. Many of these miRNAs are conserved across species [50]. That finding made AF the source of choice for MSCs in the current study.

Another possible mechanism of action is through MSCs trans-differentiation through their capability of homing to damaged tissues [51]. One study demonstrated that MSCs engrafted and differentiated to type II alveolar cells in the lung, and their behavior was influenced by the injurious environment [51]. Furthermore, after recruitment to site of injury, AF-MSCs interact with multiple immune cells, such as T and B lymphocytes, natural killer cells and dendritic cells, thus their powerful immunomodulatory properties [52, 53]. Additionally, they possess major anti-inflammatory (through secreting IL-4, IL-6, IL-10, IFN-γ), anti-apoptotic and anti-fibrotic properties [9, 54–56].

## Conclusion

Prolonged intermittent hyperoxia induces lung fibrosis, which is thought to be secondary to lung injury with excessive ROS production. In this study using an animal model of hyperoxia-induced lung fibrosis, using AF-MSCs as prophylaxis prior to the occurrence of fibrosis, preserved histological, physiological as well as biochemical parameters.

Furthermore, treatment with AF-MSCs markedly improved the histological structure of the lung alveoli, detected by both light and electron microscopes. Additionally, it improved pulmonary function tests and reduced ROS production. Moreover, it decreased inflammatory cells infiltration of lung parenchyma with significant decrease in IL-6, together with decreased fibrotic marker RhoA, subsequently reducing collagen fibrils observed with hyperoxia as evident by reduced the area percentage of collagen deposition.

## Limitations of the current study

Despite the satisfactory results obtained in the current study, long term follow-up of rats was not applied to monitor whether this improvement will be life-long and will affect the rats’ longevity, and also to monitor any possible latent side effects. Moreover, it would be of utmost benefit to explore whether multiple stem cell injections would have added up to these satisfactory results, or merely a single injection is all it takes as done in the present study. These issues deserve more attention in the future study designs.

## Supplementary Information

**Additional file 1**. Supplementary information file (S1) for figure 7: Full length original western blot analysis for IL-6 and RhoA in all studied experimental groups. Beta actin was used for normalization.

## Data Availability

The datasets used and/or analyzed during the current study are available from the corresponding author on reasonable request.

## Abbreviations

AF-MSCs: Amniotic fluid mesenchymal stem cells
IL6: Interleukin-6
RhoA: Ras homolog family member A
LM: Light microscope
EM: Electron microscope
CG: Control group
HG: Hyperoxia group
CFM-TG: Cell-free media-treated group
SC-PG: Stem cell- prophylactic group
SC-TG: Stem cell- treated group
Unt-G: Untreated group
DIC: Differential interference contrast
TV: Tidal volume
MRV: Minute respiratory volume
RR: Respiratory rate
FVC: Forced vital capacity
FEV1: Forced expiratory volume 1

## References

[1] Coronavirus disease (COVID-19). https://www.who.int/emergencies/diseases/novel-coronavirus-2019?gclid=CjwKCAjwgviIBhBkEiwA10D2j2lSvvnoCUAcOuPALpY-4G8qQ3k4RJd2WtlmsPh6797H6epLemND_hoCb4cQAvD_BwE.

[2] Hanidziar D, Robson SC (2021). Hyperoxia and modulation of pulmonary vascular and immune responses in COVID-19. Am J Physiol Lung Cell Mol Physiol.

[3] Kondrikov D, Caldwell RB, Dong Z, Su Y (2011). Reactive oxygen species-dependent RhoA activation mediates collagen synthesis in hyperoxic lung fibrosis. Free Radic Biol Med.

[4] Rai DK, Sharma P, Kumar R (2021). Post covid 19 pulmonary fibrosis. Is it real threat?. Indian J Tuberc.

[5] Pala Cifci S, Urcan Tapan Y, Turemis Erkul B, Savran Y, Comert B. The impact of hyperoxia on outcome of patients treated with noninvasive respiratory support. Can Respir J. 2020;2020. 10.1155/2020/3953280 PMC7225844 32454913

[6] Amarelle L, Quintela L, Hurtado J, Malacrida L. Hyperoxia and lungs: what we have learned from animal models. Front Med. 2021;8:606678. 10.3389/fmed.2021.606678 PMC7985075 33768102

[7] Chu DK, Kim LH, Young PJ, Zamiri N, Almenawer SA, Jaeschke R, Szczeklik W, Schünemann HJ, Neary JD AW, Chu DK, Kim LH-Y, Young PJ, Zamiri N, Almenawer SA, et al. Mortality and morbidity in acutely ill adults treated with liberal versus conservative oxygen therapy (IOTA): a systematic review and meta-analysis. Lancet. 2018;391(10131):1693–705. 10.1016/S0140-6736(18)30479-3 29726345

[8] Savickiene J, Treigyte G, Baronaite S, Valiuliene G, Kaupinis A, Valius M, et al. Human amniotic fluid mesenchymal stem cells from second- and third-trimester amniocentesis: differentiation potential, molecular signature, and proteome analysis. Stem Cells Int. 2015;2015. 10.1155/2015/319238 PMC4553339 26351462

[9] Tatullo M, Gargiulo IC, Dipalma G, Ballini A, Inchingolo ADAM, Paduanelli G, et al. Stem cells and regenerative medicine. In: Stephen T. Sonis AV, editor. Translational systems medicine and oral disease 2020. 10.1016/B978-0-12-813762-8.00017-7.

[10] Nelson JF, Felicio LS, Randall PK, Sims C FC, Nelson JF, Felicio LS, Randall PK, Sims C, Finch CE. A longitudinal study of estrous cyclicity in aging C57BL/6J mice: I. Cycle frequency, length and vaginal cytology. Biol Reprod. 1982;27(2):327–339. 10.1095/biolreprod27.2.327 6889895

[11] Wen ST, Chen W, Chen HL, Lai CW, Yen CC, Lee KH et al. Amniotic fluid stem cells from EGFP transgenic mice attenuate hyperoxia-induced acute lung Injury. PLoS ONE. 2013;8:e75383. 10.1371/journal.pone.0075383 PMC3770548 24040409

[12] Gholizadeh-Ghalehaziz S, Farahzadi R, Fathi EPM (2015). A mini overview of isolation, characterization and application of amniotic fluid stem cells. Int J Stem Cells.

[13] Wang M, Li H, Si J, Dai J, Shi J, Wang X (2017). Amniotic fluid-derived stem cells mixed with platelet rich plasma for Restoration of rat alveolar bone Defect. Acta Biochim Biophys Sin.

[14] Al-Husseiny F, Sobh MA, Ashour RH, Foud S, Medhat TE, Gilany AH (2016). Amniotic fluid-derived mesenchymal stem cells in, cut short the acuteness of cisplatin-induced nephrotoxicity Sprague–Dawley rats. Int J Stem Cells.

[15] Pochampally R (2008). Colony forming unit assays for MSCs. Methods Mol Biol.

[16] Abolgheit S, Abdelkader S, Aboushelib M, Omar EMR (2021). Bone marrow-derived mesenchymal stem cells and extracellular vesicles enriched collagen chitosan scaffold in skin wound healing (a rat model). J Biomater Appl.

[17] CellTracker^TM^ CM-DiI Dye. https://www.thermofisher.com/order/catalog/product/C7000.

[18] Sample preparation for fluorescence microscopy: an introduction - concepts and tips for better fixed sample imaging results | July 28, 2015. https://www.biotek.com/resources/white-papers/sample-preparation-for-fluorescence-microscopy-an-introduction-concepts-and-tips-for-better-fixed-sample-imaging-results/.

[19] Khalifa Y, Mourad G, Stephanos WM, Omar S, Mehanna R (2019). Bone marrow-derived mesenchymal stem cell potential regression of dysplasia associating experimental liver fibrosis in albino rats. Biomed Res Int.

[20] Limjunyawong N, Mitzner W, Horton MR (2014). A mouse model of chronic idiopathic pulmonary fibrosis. Physiol Rep.

[21] Drury RWE (1980). Light microscope and slide preparation, Carleton’s histological technique.

[22] Pereira-Fantini PM, Oakley RB, Tingay DG, McCall KE, Perkins EJ, Sourial M (2019). Gestational age influences the early microarchitectural changes in response to mechanical ventilation in the preterm lamb lung. Front Pediatr.

[23] Graham R, Gray T, Bancroft J SA. Electron microscopy 2: practical procedures. Theory and practice of histological techniques. 4th ed. New York: Churchill-Livingstone.; 1996.

[24] Burnette WN (1981). “Western blotting”: electrophoretic transfer of proteins from sodium dodecyl sulfate–polyacrylamide gels to unmodified nitrocellulose and radiographic detection with antibody and radioiodinated protein A. Anal Biochem.

[25] Waterborg JH, Walker JM (2009). The Lowry method for protein quantitation. The protein protocols handbook.

[26] Koracevic D, Koracevic G, Djordjevic V, Andrejevic SCV (2001). Method for the measurement of antioxidant activity in human fluids. J Clin Pathol.

[27] Ohkawa H, Ohishi N, Yagi K (1979). Assay for lipid peroxides in animal tissues by thiobarbituric acid reaction. Anal Biochem.

[28] Kotz S, Read CB, Balakrishnan N, Vidakovic BJN (2006). Encyclopedia of statistical sciences.

[29] Asfar P, Singer M, Radermacher P (2015). Understanding the benefits and harms of oxygen therapy. Intensive Care Med.

[30] Vyas-Read S, Wang W, Kato S, Colvocoresses-Dodds J, Fifadara NH, Gauthier TW (2014). Hyperoxia induces alveolar epithelial-to-mesenchymal cell transition. Am J Physiol Lung Cell Mol Physiol.

[31] Davies J, Karmouty-Quintana H, Le TT, Chen NY, Weng T, Luo F (2014). Adenosine promotes vascular barrier function in hyperoxic lung injury. Physiol Rep.

[32] Khoubnasab Jafari M, Ansarin K, Jouyban A (2015). Comments on “use of malondialdehyde as a biomarker for assesing oxidative stress in different disease pathologies: a review”. Iran J Public Health.

[33] Li H, Wang G, Lin S, Wang C, Zha J (2019). Loss of interleukin-6 enhances the inflammatory response associated with hyperoxia-induced lung injury in neonatal mice. Exp Ther Med.

[34] Voiriot G, Razazi K, Amsellem V, Tran Van Nhieu J, Abid S, Adnot S, et al. Interleukin-6 displays lung anti-inflammatory properties and exerts protective hemodynamic effects in a double-hit murine acute lung injury. Respir Res. 2017;18(1):1–14. 10.1186/s12931-017-0553-6 PMC5397701 28424078

[35] Tuder RM, Hunt JM, Schmidt EP (2011). Hyperoxia and apoptosis: Too much of a good thing?. Am J Respir Crit Care Med.

[36] Kallet RH, Matthay MA (2013). Hyperoxic acute lung injury. Respir Care.

[37] Brandt JP, Mandiga P. Histology, alveolar cells. StatPearls. StatPearls Publishing; 2021 [cited 2021 Nov 7]. https://www.ncbi.nlm.nih.gov/books/NBK557542/.

[38] You K, Xu X, Fu J, Xu S, Yue X, Yu Z, Xue X (2012). Hyperoxia disrupts pulmonary epithelial barrier in newborn rats via the deterioration of occludin and ZO-1. Respir Res.

[39] Hemnes AR, Zaiman A, Champion HC (2008). PDE5A inhibition attenuates bleomycin-induced pulmonary fibrosis and pulmonary hypertension through inhibition of ROS generation and RhoA/Rho kinase activation. Lung Cell Mol Physiol.

[40] McElroy MC, Kasper M (2004). The use of alveolar epithelial type I cell-selective markers to investigate lung injury and repair. Eur Respir J.

[41] Matthew A. Wallig EBJ. Morphologic manifestations of toxic cell injury. In: Wallig MA, Haschek WM, Rousseaux CGBB, editor. Fundamentals of toxicologic pathology. 3rd ed. Academic Press; 2018. p. 59–81.

[42] Raftopoulou M, Hall A (2004). Cell migration: Rho GTPases lead the way. Dev Biol.

[43] MacKay CE, Shaifta Y, Snetkov VV, Francois AA, Ward JP, Knock GA (2017). ROS-dependent activation of RhoA/Rho-kinase in pulmonary artery: role of Src-family kinases and ARHGEF1. Free Radic Biol Med.

[44] Loukogeorgakis SP, De Coppi P (2017). Concise review: amniotic fluid stem cells: the known, the unknown, and potential regenerative medicine applications. Stem Cells.

[45] Devine SM, Cobbs C, Jennings M, Bartholomew A, Hoffman R (2003). Mesenchymal stem cells distribute to a wide range of tissues following systemic infusion into nonhuman primates. Blood.

[46] Chang YS, Ahn SY, Yoo HS, Sung SI, Choi SJ, Oh WI PW. Mesenchymal stem cells for bronchopulmonary dysplasia: phase 1 dose-escalation clinical trial. J Pediatr. 2014;164(5). 10.1016/j.jpeds.2013.12.011 24508444

[47] Ahn SY, Chang YS, Kim JH, Sung SIPW (2017). Two-year follow-up outcomes of premature infants enrolled in the phase I Trial of mesenchymal stem cells transplantation for bronchopulmonary dysplasia. J Pediatr.

[48] Hemnes AR, Zaiman A, Champion HC, Hemnes AR, Zaiman ACH, Hemnes AR, Zaiman A (2008). PDE5A inhibition attenuates bleomycin-induced pulmonary fibrosis and pulmonary hypertension through inhibition of ROS generation and RhoA/Rho kinase activation. Am J Physiol Cell Mol Physiol.

[49] Gong M, Yu B, Wang J, Wang Y, Liu M, Paul C (2017). Mesenchymal stem cells release exosomes that transfer miRNAs to endothelial cells and promote angiogenesis. Oncotarget.

[50] Antounians L, Catania VD, Montalva L, Liu BD, Hou H, Chan C, et al. Impaired fetal lung development can be rescued by administration of extracellular vesicles derived from amniotic fluid stem cells. bioRxiv. 2020. 10.1101/2020.08.07.240408.

[51] Huang K, Kang X, Wang X, Wu S, Xiao J, Li Z (2015). Conversion of bone marrow mesenchymal stem cells into type II alveolar epithelial cells reduces pulmonary fibrosis by decreasing oxidative stress in rats. Mol Med Rep.

[52] Zakaria D, Zahran N, Arafa S, Mehanna R, Abdel-Moneim R. Histological and physiological studies of the effect of bone marrow-derived mesenchymal stem cells on bleomycin induced lung fibrosis in adult albino rats. Tissue Eng Regen Med. 2020. 10.1007/s13770-020-00294-0 PMC7579902 33090319

[53] Yang S, Liu P, Jiang Y, Wang Z, Daiand H, Wang C. Therapeutic applications of mesenchymal stem cells in idiopathic pulmonary fibrosis. Front Cell Dev Biol. 2021. 10.3389/fcell.2021.639657 PMC7985078 33768094

[54] Ju X, Li Z, Gong X, Li D, Yang X, Shi Q. Intratracheal transplantation of amnion-derived mesenchymal stem cells ameliorates hyperoxia-induced neonatal hyperoxic lung injury via aminoacyl-peptide hydrolase. Int J Stem Cells. 2020. 10.15283/ijsc19110. 10.15283/ijsc19110 PMC7378897 32323511

[55] Mirershadi F, Ahmadi M, Rezabakhsh A, Rajabi H, Rahbarghazi R, Keyhanmanesh R (2020). Unraveling the therapeutic effects of mesenchymal stem cells in asthma. Stem Cell Res Ther.

[56] Keyhanmanesh R, Rahbarghazi R, Ahmadi M (2018). Systemic transplantation of mesenchymal stem cells modulates endothelial cell adhesion molecules induced by ovalbumin in rat model of asthma. Inflamm.

