# Supplementary material for: Potential effect of amniotic fluid-derived stem cells on hyperoxia-induced pulmonary alveolar injury
Source: Stem Cell Res Ther. 2022 Apr 4;13:145. doi: 10.1186/s13287-022-02821-3 (PMC8978174; doi:10.1186/s13287-022-02821-3)
Supplement: Supplementary file 1 — Additional file 1. Supplementary information file (S1) for figure 7: Full length original western blot analysis for IL-6 and RhoA in all studied experimental groups. Beta actin was used for normalization. [file 13287_2022_2821_MOESM1_ESM.docx]

**Potential Effect Of Amniotic Fluid-Derived Stem Cells On Hyperoxia-Induced Pulmonary Alveolar Injury**

Amany Solaiman^1^, Radwa A. Mehanna^2,3^, Ghada A. Meheissen^1,*^, Soha Elatrebi^4^, Rasha Said^5^, Nahed H. Elsokkary^2^

1. Histology and Cell Biology Department, Faculty of Medicine, Alexandria University, Egypt
2. Medical Physiology Department, Faculty of Medicine, Alexandria University, Egypt
3. Center of Excellence for Research in Regenerative Medicine and its Applications CERRMA, Faculty of Medicine, Alexandria University, Egypt
4. Clinical pharmacology Department, Faculty of Medicine, Alexandria University, Egypt
5. Biochemistry Department, Faculty of Medicine, Alexandria University, Egypt

* Corresponding author

*Dr. Ghada Ahmed Meheissen*

*Lecturer of Histology and Cell Biology,*

*Faculty of Medicine, University of Alexandria, Egypt.*

*Email:* [*Ghada.mohisen@alexmed.edu.eg*](mailto:Ghada.mohisen@alexmed.edu.eg)

*Address: Faculty of Medicine, Dr Fahmi Abdelmeguid St., Al. Mowassat Campus, Alexandria 21561, Egypt.*

*Phone #: 002 01558857358*

*ORCID #: 0000-0001-5910-8292*


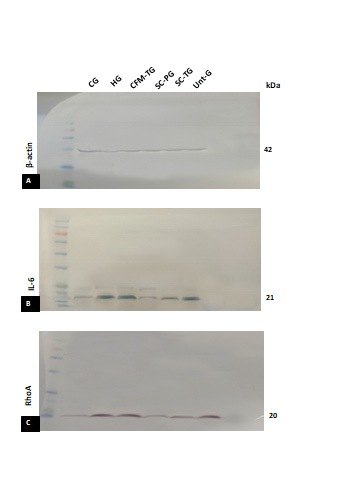


**Figure S1** **Full length original western blot analysis for (B) IL-6 and (C) RhoA** in all studied experimental groups where β- actin **(A)** was used for normalization.

IL-6 and RhoA are relatively overexpressed in the HG, CFM-TG and Unt-G groups while prophylaxis and treatment by stem cells underexpresses them in the SC-PG and SC-TG respectively.

Abbreviations: CG; control group, HG; Hyperoxia group, CFM-TG; Cell-free media-treated group, SC-PG; Stem cells-prophylactic group, SC-TG; Stem cells-treated group, Unt-G Untreated group, IL-6; interleukin 6, RhoA; Ras homolog family member A
